# Does Electrification Spur the Fertility Transition? Evidence From Indonesia

**DOI:** 10.1007/s13524-015-0420-3

**Published:** 2015-08-26

**Authors:** Michael Grimm, Robert Sparrow, Luca Tasciotti

**Affiliations:** Department of Economics, University of Passau, Innstraße 29, 94032 Passau, Germany; Erasmus University Rotterdam, P.O. Box 29776, 2502 LT The Hague, The Netherlands; IZA, Bonn, Germany; Arndt-Corden Department of Economics, Crawford School of Public Policy, Australian National University, Canberra, ACT 2601 Australia

**Keywords:** Fertility, Fertility transition, Family planning, Electrification, Television

## Abstract

We analyze various pathways through which access to electricity affects fertility in Indonesia, using a district difference-in-difference approach. The electrification rate increased by 65 % over the study period, and our results suggest that the subsequent effects on fertility account for about 18 % to 24 % of the overall decline in fertility. A key channel is increased exposure to television. Using in addition several waves of Demographic and Health Surveys, we find suggestive evidence that increased exposure to TV affects, in particular, fertility preferences and increases the effective use of contraception. Reduced child mortality seems to be another important pathway.

**Electronic supplementary material:**

The online version of this article (doi:10.1007/s13524-015-0420-3) contains supplementary material, which is available to authorized users.

## Introduction

In most developing countries, fertility is declining, albeit at different speeds within and across countries. The debate about the causes of that heterogeneity and the very slow progress in some areas is typically also a debate about whether family planning or economic development is more effective in triggering the fertility transition (Bongaarts 2011; Bongaarts and Casterline 2012; Casterline and Sinding 2000; Cleland et al. 2006; Molyneaux and Gertler 2000; Pritchett 1994). Electrification may play a role for both. On the one hand, electrification comes with access to television (TV) and other modern media, which may improve the access to information about contraception and diffuse new norms and role models. On the other hand, because electrification is a driver of economic development, it can affect the direct and indirect costs of having children and therefore also can affect fertility choices.

In this article, we explore the effect of electrification on fertility, including some potentially relevant channels, for the case of Indonesia. Indonesia’s fertility has significantly declined since the 1970s. Whereas the total fertility rate (TFR) stood at 5.6 children per women at the end of the 1960s, it declined to 4.1 in 1985, 2.9 in 1993, and then to 2.6 in 2010, albeit with substantial variations in the speed and timing of the decline across regions and urban and rural areas (BPS 1989, 1995, 2008, 2013). The media have often attributed this decline to the dramatic rise in contraceptive use and an associated increase in the age of marriage. Indonesia introduced family planning programs in 1970, expanding rapidly throughout the country and later accompanied by an intense family planning campaign disseminated via radio and TV, among other pathways (Hull and Hull 2005). The percentage of women using contraception rose from 27 % in 1980 to 43 % in 2010. Over that same period, the median age at first marriage for ever-married women aged 25 to 49 increased from about 17 to 20 years (BPS 1989, 2013). However, a number of studies have also highlighted the effects of expanding women’s education and labor market opportunities, which may have been more important drivers of fertility decline than family planning (Angeles et al. 2005; Gertler and Molyneaux 1994; Hull and Hatmadji 1990; Hull et al. 1977; McNicoll and Singarimbun 1983; Molyneaux and Gertler 2000; Pitt et al. 1993; World Bank 1990). The share of households with an electricity connection also increased substantially over this period—between 1993 and 2010 alone, from 58 % to 95 %—which may have influenced fertility through enhanced diffusion of contraceptive knowledge and norms as well as serving as a driver of economic development. In both cases, electrification would imply second-order effects that might easily be overseen in a standard cost-benefit analysis of publicly funded electricity grid expansion.

The effect of electrification on fertility has already received some attention in the literature. Studies looking at this relationship in cross-sectional data have typically found a negative correlation but with some interesting variation (Cornwall and Robinson 1988; Harbison and Robinson 1985; Herrin 1979; Peters and Vance 2011). Cornwall and Robinson (1988) pointed to differences in southern versus nonsouthern states in the United States. Peters and Vance (2011) found contrasting patterns for urban and rural areas of Côte d’Ivoire. Two more recent studies used panel data and employed an identification strategy that is close to ours. Potter et al. (2002) exploited geographic variation in electrification in Brazil for the period 1960–1990 in a fixed-effects analysis, confirming the negative effects of expanding electricity connection on fertility found in cross-sectional data. Bailey and Collins (2011) looked at the United States during the period 1940–1960 and also used variation across time and space. They focused on electric appliance ownership and access to electric service during early adulthood, finding a negative effect of both factors on fertility, which (according to the authors) rejects the hypothesis that rapid advancement in household technology caused the Baby Boom. We rely on a similar strategy to identify the effect of electrification on fertility, but we also try to account for a larger set of time-varying and potentially confounding factors, and we use more waves of data. Another closely related strand of the literature has focused on temporary power outages instead of permanent electrification. Burlando (2013) and (Fetzer et al. 2013) are two cases in point: both found relatively strong positive effects of outages on fertility. Our results are largely in line with these previous empirical studies: we find that the increase in the electrification rate contributes substantially to the fertility decline between 1993 and 2010. An innovation of our study is that we also probe into some of the channels through which electrification affects fertility. For our analysis, we use a unique data set combining annually repeated cross-sectional household survey data with village census information to construct a panel for 261 districts, covering the period 1993–2010. The district panel allows for a district-level analysis of the causal relationship between regional expansion of the electricity grid and changes in the fertility rate, as well as the transmission channels, while controlling for district and time fixed effects. In addition, the data include a large set of variables that capture sociodemographic change and also economic and infrastructure development in districts. Although our data set allows us to control for many aspects that previous studies could not capture, endogeneity might still be an issue in such a framework because unobservable time-varying characteristics could determine both the connection of a village to the grid and fertility choices. We test the robustness of our findings by instrumenting village connection by the proximity of power plants conditional on a large set of controls as well as district fixed effects. This approach can work as long as the placement of power plants is determined by geographic features but is uncorrelated with unobserved time-varying shocks that are also correlated with fertility choices. The fixed-effects estimates are robust to including the control variables as well as the IV approach, suggesting that unobserved time-dependent and district-specific shocks are unlikely to drive the results. Increased exposure to TV and reduced child mortality rates appear to be key pathways through which access to electricity affects fertility.

## Theoretical Framework

Various channels exist by which electrification could potentially affect fertility decisions and fertility outcomes. In this section, we provide a short structured discussion of these channels inspired by the standard Beckerian model of the demand for children (see, e.g., Becker 1981; Becker and Lewis 1973; Willis 1973) as well as supply-side theory. We organize this discussion around three aspects: (1) the direct and indirect costs of children, (2) access to modern media and fertility preferences as well as (3) the quality of health care.

### Electrification and the Direct and Indirect Costs of Children

Access to electricity may influence the shadow price of children by affecting both the direct and indirect costs of raising children. The direct costs may increase if electrification improves the productivity on the labor market of the main caregiver, and hence, the opportunity cost of time for raising children. The pure income effect could, in principle, be fertility-increasing if children are considered normal goods. At the same time, electrification may reduce the costs of home production, including raising children, given that electric appliances can be used to prepare food, wash clothes, or clean the house. However, electrification may also affect the productivity of child labor, and subsequently, the indirect cost of raising children. This result is particularly relevant in rural areas where many children help their family on the farm. The direction in which electricity changes the returns to children will depend on whether electricity is a complement or a substitute to child labor. We expect the substitution effect to dominate in Indonesia, given that more than two-thirds of child labor in the age group 10–15 years takes place in agriculture (Kis-Katos and Sparrow 2011), where a technological development is likely to substitute labor.

The direction of the total price effect on fertility depends then on the net effect resulting from the direct and indirect effects on the shadow price of children. Empirically, this implies investigating the effect of access to electricity on fertility directly and on men’s, women’s, and children’s wages and on their labor market participation. For South Africa, Dinkelman (2011) found relatively strong effects of household electrification on women’s labor market participation. Barron and Torero (2014) found somewhat smaller effects for El Salvador. For India, Van de Walle et al. (2013) found effects on only the female labor supply for casual work. Electrification may also open up new business opportunities or increase the productivity of existing businesses, as found by van de Walle et al. (2013), Lipscom et al. (2013), Khandker et al. (2013), and Chakravorty et al. (2013). However, in many other studies—particularly in relatively poorer and more remote areas where many other constraints prevail—such effects could not be observed, providing a rather mixed evidence base (Bernard 2012).

### Access to Modern Media and Fertility Preferences

Three aspects are particularly relevant here—that the exposure to modern media may (1) affect norms, (2) provide information, for example about contraception, and (3) affect the allocation of time. We briefly discuss each aspect.

Electrification may affect the marginal utility of children through the exposure to TV and other electronic media that broadcast new norms, which may alter fertility preferences. That is, even at constant income and prices, the desired number of children may decrease, conforming to the fertility preferences depicted by modern households in TV shows. For example, La Ferrara et al. (2012) showed that the exposure to soap operas, particularly those depicting women of lower socioeconomic status, lowered fertility through the role models that were portrayed. Jensen and Oster (2009) found similar effects for India (for a review, see Basten 2009).

Obviously, even if the desired number of children is not affected, the exposure to new media may still affect the actual number of children if more information about family planning services helps to reduce unwanted births by reducing the cost of not having a child (Bongaarts 1993). In this case, we are back to the conduit that we started with: namely, a change in the direct cost of children.

Modern media may also have an effect on the opportunity cost of time and thus the time needed to “produce” a child, if time spent watching TV reduces the time available for physical intimacy. Through this channel the availability of TV would further lower fertility.

### Electrification and the Quality of Health Care

If electrification improves the quality and accessibility of health care—for example, because new technologies can be used or simply because standard treatments can also be undertaken in the absence of daylight—then survival chances can be thought of as partly determined by access to electricity. Obviously, electricity-driven access to modern media could also provide information relevant to children’s health, such as information about the importance of vaccinations, micronutrients, or preventive measures against diarrhea.

Assuming that the increase in survival chances is unrelated to income, prices, and preferences, the resulting increase in parents’ number of surviving children can be interpreted as an exogenous shift in the biological supply of children (Easterlin and Crimmins 1985; Schultz 1997). This shift reduces the cost of producing a survivor while also reducing the number of births needed to have a survivor. If parents’ demand for surviving children is price-inelastic and the cost per surviving child decreases in proportion to the increase in the survival rate, then parents should theoretically respond by reducing the number of births because they need fewer births to attain a given number of survivors (Sah 1991; Schultz 1997). If, in addition, parents are risk-averse, the need for “hoarding” declines as well, which will further reduce the number of births (Schultz 1997).

In the next section, we present the data that allow us to test some of these channels empirically and provide more information about the context.

## Data and Context

In this article, we use four sources of data. The core of our analysis draws on the annual Indonesian national socioeconomic household survey (SUSENAS) from 1993 to 2010, and the village census (PODES) for 1996, 2000, 2003, 2006, and 2008. Both the SUSENAS and PODES are conducted by Statistics Indonesia (*Badan Pusat Statistik* (BPS)). In addition, we use data from the Indonesian Demographic and Health Surveys (DHS) conducted in 1991, 1994, 1997, 2002/2003, and 2007. Finally, we use a data set retrieved from the Indonesian National Electricity Provider PLN (*PT Perusahaan Listrik Negara*) containing information on all power plants with a capacity of more than 1 megawatt (MW); their exact location; the date they started operation; and, if applicable, the date the plant was shut down. Between 1993 and 2010, 133 power plants of that size were in operation.

The annual cross-sectional data that can be drawn from the SUSENAS are representative at the district level and can be used to construct a district panel, yielding a balanced panel of 261 districts for 16 years. We have to drop the districts in the provinces of Aceh, Papua, and the Maluku Islands, which were not included in the survey in some years because of violent conflicts. All other districts are represented in each year. Because a number of districts split up over time, particularly after 2001, we apply a geographic definition of districts that is consistent over time. To this end, we use the 1993 districts definition for all years, combining districts that split up to form the original parent district.^1^

In all years except 2005, the SUSENAS included a question on the source of lighting for the household, which we use as indication of electricity connection. Based on this information, we construct a variable measuring the district-specific share of households reporting electricity as the main source of lighting. Information on fertility is based on questions to females aged 10 years and older, regarding the number of biological children. We construct fertility rates as the average number of live births per woman. Given that the number of children a woman has depends strongly on age, we control in all our regressions for the district-specific age composition. Moreover, we also estimate the main regressions separately for different age groups to see whether our results differ across age (or cohorts). A problem with our fertility measure could arise if electrification affects the timing of birth: that is, if electrification leads women to have their children later without altering the total number of children they have. In this case, we would overestimate the effect of electrification on completed fertility. However, we see no particular reason why electrification should affect only the timing of births. Moreover, we also look at fertility preferences (i.e., the total number of desired children), which is not affected by timing effects.

The survey further includes an array of socioeconomic variables, including education (highest level completed), use of contraceptives, labor market participation, and detailed household spending. We use these household expenditures as a proxy for income in the analysis. For the years 1993–1998, the questionnaire also included a module on mass media, asking respondents whether they have watched TV, listened to radio, or read a newspaper in the previous week, thus allowing us to test whether electrification affects fertility through the exposure to new media. Except for the most recent years, however, the survey collected no data regarding the use of computers or cell phones.

The PODES village census covers all villages (*desa*) and urban precincts (*kelurahan*) in Indonesia, collecting information from the village or precinct head. The village census is aggregated at the district level using village population weights and then merged to the SUSENAS district panel. The PODES village census provides information on infrastructure and economic development. We found consistent questions across PODES waves regarding economic structure (agriculture as the main activity), presence of a market with a (semi-) permanent building or larger shopping complex, the quality of roads (whether the majority of traffic uses an asphalt road), source of clean drinking water (pump or piped water), health care providers (maternity clinic/hospitals, health centers, and village maternity posts) and schools in the village.

The DHS data are used to probe the hypotheses regarding desired fertility and child mortality that cannot be tested with the SUSENAS data. To collect information on desired fertility, the DHS survey asks all women aged 15–49 how many children they would want if they could start afresh. Interpretation of answers to this kind of question has been subject of controversy. The main point of criticism is that the response may reflect the preferences of the current context in which women live and the associated social pressure, rather than intrinsic fertility preferences. However, BPS and Macro International (BPS 2008) argued that this criticism may not be relevant for Indonesia, where family planning is widely supported and used, and social pressure is likely to be less of an issue. The main drawback of the DHS data is that they are not suitable for building a district panel because the sample is not representative at the district level. Hence, we cannot claim to identify causal effects for the analysis based on the DHS data.

**Fig. 1:**
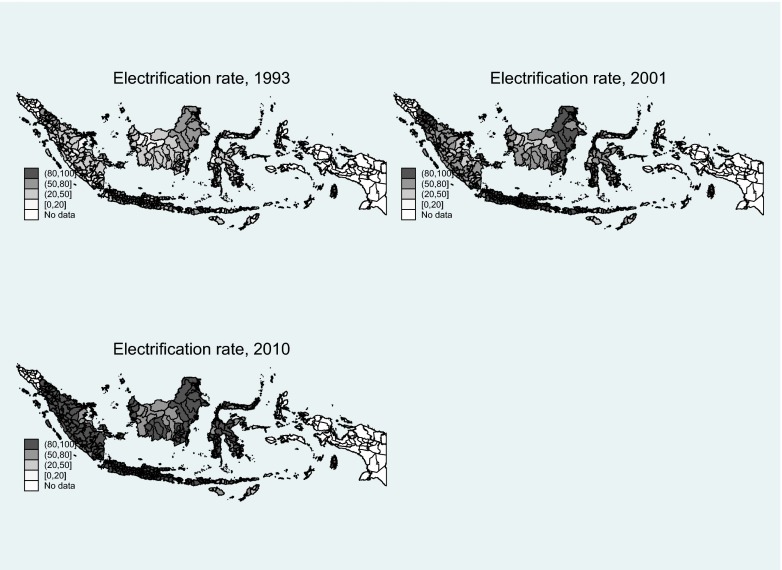
Rollout of the electricity grid across space. *Source:* SUSENAS household surveys, own representation

Descriptive statistics for the variables at the district level are presented in Table 1. The electrification rate increased steadily from 1993 to 2010, particularly during the 1990s. According to the SUSENAS data, the share of the population with access to electricity increased from 57.6 % in 1993 to 94.9 % in 2010. The data show that most electricity connections are to the national electricity grid of the state utility PLN, with only 2 % to 4 % of households having a connection to an alternative source: for example, diesel generators, car batteries, micro-hydro generators, and solar panels. The temporal rollout of access to electricity across the archipelago is illustrated in Fig. 1, showing that in 1993, the electrification rate was highest on Java and relatively low on most other islands. This expansion was also one of the objectives anchored in Indonesia’s five-year development plans (*Repelita*), which followed both cost considerations (i.e., the proximity of a village to the existing grid and market size) and equity considerations (i.e., the development plans explicitly favored particularly poor and remote locations). Locations that could not be reached through the grid were typically supplied electricity using diesel generators or other off-grid solutions, mostly also by PLN (World Bank 2003). Given these two objectives, in practice, targets and programs have evolved in a rather ad hoc manner. The involvement of many bilateral and multilateral donors, each with its own priorities, further affected the actual rollout (Munasinghe 1988; World Bank 2004). By 2001, the electrification rate exceeded 50 % on almost all islands, with North Sumatra and East Kalimantan exceeding 80 %. By 2010, all districts except for a few other remote rural areas on Sumatra, West and Central Kalimantan, and Sulawesi realized electrification rates of at least 80 %.

**Table 1:** Descriptive statistics district panel, 261 districts,^a^ 1993–2010

Variable	Number of Observations	Mean	SD
SUSENAS			
Age	4,697	27.95	2.82
Female share	4,697	0.50	0.01
Household size	4,697	4.89	0.53
Rural share	4,697	0.61	0.32
Electricity coverage^b^	4,436	0.79	0.23
PLN	4,436	0.75	0.25
Other source (off grid)	4,436	0.04	0.07
Composition of female population			
Share aged 15–19	4,697	0.18	0.03
Share aged 20–24	4,697	0.16	0.03
Share aged 25–29	4,697	0.16	0.02
Share aged 30–34	4,697	0.15	0.02
Share aged 35–39	4,697	0.14	0.02
Share aged 40–44	4,697	0.12	0.02
Share aged 45–49	4,697	0.10	0.02
Fertility rate (average number of live births)			
Women aged 15–49	4,697	1.88	0.34
Women aged 15–24	4,697	0.29	0.11
Women aged 25–34	4,697	1.81	0.40
Women aged 35–49	4,697	3.49	0.74
Contraceptives used	4,697	0.38	0.11
Traditional contraceptives used	4,697	0.005	0.01
Real monthly per capita expenditure (*Rupiah*)	4,697	287,964	263,325
Child work	4,697	0.10	0.08
Female work	4,697	0.50	0.13
Media exposure in previous week (1993–1998 only)			
TV	1,566	0.61	0.26
Radio	1,566	0.51	0.21
Newspaper	1,566	0.19	0.15
Highest education completed by women aged 15–49			
None	4,697	0.34	0.14
Primary	4,697	0.31	0.08
Junior secondary	4,697	0.17	0.07
Senior secondary	4,697	0.13	0.08
Higher	4,697	0.05	0.05
PODES^c^			
Agriculture main activity in village	1,296	0.69	0.32
Market with (semi-) permanent building in village	1,296	0.29	0.17
Shopping complex in village	1,296	0.27	0.21
Majority of traffic on asphalt road in village	1,296	0.73	0.22
Drinking water piped/pump in village	1,296	0.33	0.29
Number of primary schools in village	1,296	4.32	2.72
Number of junior secondary schools in village	1,296	1.01	0.87
Number of senior secondary schools in village	1,296	0.59	0.67
Number of maternity clinics/hospitals in village	1,296	0.16	0.18
Number of health centers in village	1,296	0.24	0.17
Number of village maternity posts in village	1,296	0.33	0.25

*Source*: SUSENAS household surveys and PODES village census.

^a^The panel is balanced for all years except 2006, for which *N* = 260. The provinces of Aceh, Papua, and the Maluku Islands are excluded throughout.

^b^Electrification rate is missing for 2005.

^c^PODES data is available only for 1996, 2000, 2003, 2006, and 2008.

Both the level and rate of change in access to electricity also varies greatly by income level. Using per capita expenditure as a proxy for income and ranking the population into per capita expenditure quintiles, we find that only 33 % of the poorest quintile but 86.4 % of the richest quintile had access to electricity in 1993. However, the expansion of the PLN grid over the following 16 years led to a more than proportional increase for the poorest quintile. By 2008, households in the richest quintile had close to universal access (98.5 %), and those in the poorest quintile nearly caught up (88.0 %).

**Fig. 2:**
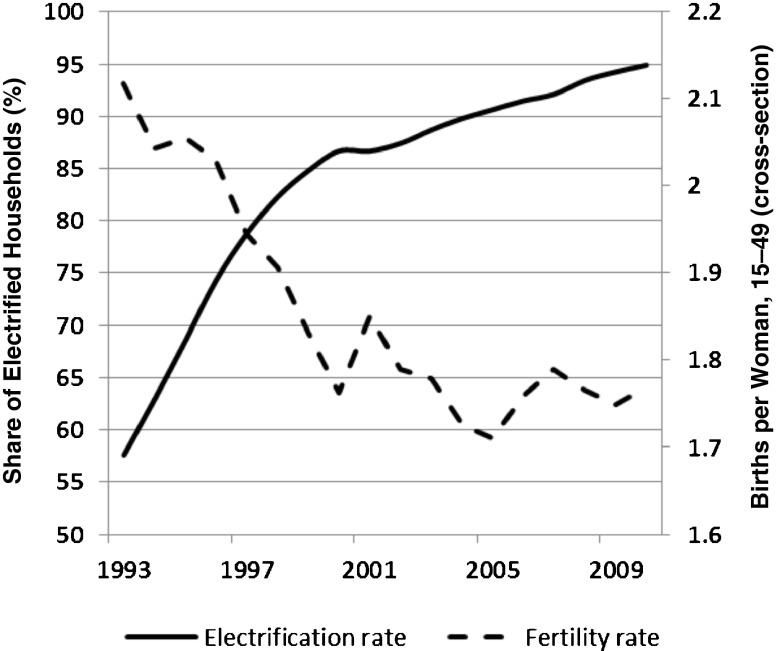
Electrification rate (%) and fertility (average number of live births per woman aged 15–49) over time, Indonesia. *Source:* SUSENAS household surveys

Figure 2 shows that over the period 1993–2008, the average number of live births per woman aged 15–49 decreased from 2.1 to 1.8. The temporary rise in fertility in 2001 most likely reflects a post-Asian crisis catch-up effect. The small increase of fertility after 2005 might be due to the reversal of the trend in the average age at marriage. Hull (2013) showed that in 2005, the age at marriage started to decline again after a long rise.

The general trend is similar to what DHS data reveal, although the level is clearly different given that we do not focus on completed fertility but instead on age-specific fertility. Another difference is that the SUSENAS data include all women between 15 and 49 years old, whereas the DHS focuses only on married women. This leads to a significant overestimation of women’s fertility in the DHS data (Hull and Hartanto 2009). In 1994, the DHS-based total fertility rate stood at 2.9 and in 2007 at 2.6, suggesting that fertility declined by about 9 % (BPS 2008). Our data yield a quite similar negative growth rate of 12.4 % over the same period. Moreover, if we condition on married, divorced, and widowed women aged 15–49, we find 2.854 children per woman for 1994 and 2.413 children per women for 2007, which is relatively close to the DHS figures.

Figure 2 shows that the aggregate time series of electrification and fertility are strongly correlated.^2^ Particularly striking is the strong increase in electrification and decline in fertility between 1993 and 2000. After 2000, the rate of change slowed for both variables. However, although the electrification rate maintained a steady increase until 2010, fertility showed strong fluctuations around a slightly declining trend. The main empirical challenge in this study will be to discern the causal relationship from this correlation.

## Empirical Specifications

### Fertility and Electrification

We model the fertility rate in a district *i* at time *t* as a linear function of the electrification rate, *e*_*i*_, in that district and a set of time-variant district characteristics, *x*_*i*_:

1 $$ f{r}_{it}=\upalpha +\upbeta {e}_{it-1}+\upgamma {x}_{it-1}+{\upeta}_i+{\updelta}_t+{\upvarepsilon}_{it}. $$

We further include district fixed effects, η_*i*_, and dummy variables for time, δ_*t*_, with the error term ε_*it*_ assumed to be a random disturbance. The electrification rate and control variables are lagged by one year, given that fertility decisions and conception occur at least nine months prior to the timing of the household survey. Given that the number of reported live births depends on the age of the woman, *x*_*i*_ includes in all regressions the district-specific age composition of women. Because it is based on live births, *fr*_*it*_ is a measure of actual fertility rather than of desired fertility. However, using the DHS data, we also investigate the determinants of desired fertility. The control variables *x*_*i*_ also include the rural population share in the district, the average educational levels of women aged 15–59, and average per capita household expenditure.

The empirical relationship between fertility and the electrification rate is prone to several sources of bias. There may be simultaneity bias given that fertility through its effect on population density may increase the demand for electricity, which in turn could increase the likelihood that a particular district will be electrified. By taking up lagged values of *e*_*i*_ and *x*_*i*_, any bias due to direct reverse causality should be eliminated. Omitted variables are another source of bias. Time-invariant characteristics, such as cultural and institutional determined factors, are controlled for by including district fixed effects. Time-variant characteristics are more difficult to deal with because we may expect potentially confounding trends in wealth as well as economic and infrastructural development in districts, which could simultaneously affect PLN grid expansion and fertility preferences. First, average district wealth is captured by average per capita household expenditure, lagged by one year. Second, we use data from the PODES village census on economic and infrastructural development characteristics of villages to conduct a sensitivity analysis for the subset of years in which the PODES was fielded. If the results are biased because of confounding economic and infrastructural development, we would expect the estimates to be sensitive to adding PODES village variables. Third, the problem of omitted time-variant factors is reduced by the fact that due to the time lag between the district averages of fertility and electrification, both variables are not computed over the same sample of women since the SUSENAS draws a new sample of households every year.

Finally, to further assess the robustness of our fixed-effects estimates, we take an instrumental variables (IV) approach (following the empirical strategy suggested by Van de Walle et al. (2013)), in which we instrument the district electrification rate by the presence of at least one power plant in the same (*p*_*it*_) or a neighboring district (*pn*_*it*_). In this case, the first-stage equation is given by

2 $$ {e}_{it}=\upvarphi +\upchi {p}_{it}+\upkappa p{n}_{it}+\upgamma {x}_{it-1}+{\upphi}_i+{\upvartheta}_t+{\upomega}_{it}, $$

where all other variables have the same notation as earlier. The key identifying assumption is that the location of power plants determines village access to electricity but does not have any direct effect on fertility other than through the supply of electricity. For instance, unobserved preferences simultaneously may determine fertility outcomes in a village and the connection of that same village to the electricity grid. We assume that the placement of a power plant in the district where the village is located, or a neighboring district, is exogenous to these preferences after district fixed effects are controlled for. Given that a key factor determining the placement of power plants is the local access to natural resources for generating electricity (rivers, coal, oil, gas, and geothermal sources), district fixed effects are expected to absorb most of the possibly confounding unobserved heterogeneity. The exogeneity assumption might be violated if in the short term, the location of power plants is influenced by local economic development within the fixed-effects setup. However, the exogeneity assumption should still be credible when it concerns the location of older power plants. In this case, we would expect that the placement of the older power plants is no longer correlated with endogenous changes, but merely with time-invariant district characteristics. We therefore apply two alternative specifications: (1) using all power plants operational in year *t* as instruments, and (2) using only those power plants that have been in operation for at least five years.

### Transmission Channels

Guided by our theoretical framework, we assess the role of three potential transmission channels through which electrification may affect fertility: (1) children’s and women’s labor market participation, (2) media exposure, and (3) child mortality. We apply a fixed-effects specification similar to that in Eq. (1). Whenever fertility is on the left hand side, we use explanatory variables lagged by one year. If intermediate variables are on the left hand side, for example children’s labor market participation, no lag is used because we expect an immediate effect. To explore the relevance of a particular channel, we first test whether electrification has a significant effect on the channel variable under study. If so, we then test whether the channel variable has a significant effect on fertility. Finally, we test whether (1) the direct effect of electricity is altered if the channel variable is included in the fertility equation, and (2) whether the channel variable is statistically significant. If the latter is the case, we take this as suggestive evidence that this particular channel is empirically relevant.

## Results

### Does Electrification Affect Fertility?

Table 2 summarizes the results estimating Eq. (1) with the district-level data for different specifications. Columns 1 and 2 show simple random- and fixed-effects models, respectively, in which we do not control for any time-variable effects except for dummy variables for year. In both cases, electricity is highly significant, but the district fixed effects appear to explain part of the correlation captured by the random-effects model. The within estimator suggests that an increase in the district coverage by 10 percentage points is associated with a reduction of the (district-level) average number of live births per woman aged 15–49 in the following year by 0.027 children, or by 1.3 %. As more control variables are added, the size of the electricity effect decreases somewhat but stays negative and statistically significantly different from zero (column 3). The electricity effect also persists if the model is estimated separately for subsamples of rural and urban households.

**Table 2:** Impact of electrification on fertility (average number of live births), women ages 15–49, 1993–2010

	All	All	All	Rural	Urban	Ages 15–24	Ages 25–34	Ages 35–49
	(1)	(2)	(3)	(4)	(5)	(6)	(7)	(8)
Electricity Coverage	–0.3227**	–0.2695**	–0.2374**	–0.1718**	–0.1826*	–0.0692**	–0.2170**	–0.1484^†^
	[0.0390]	[0.0395]	[0.0414]	[0.0497]	[0.0851]	[0.0171]	[0.0603]	[0.0811]
Year Dummy Variables	Yes	Yes	Yes	Yes	Yes	Yes	Yes	Yes
District Fixed Effects	No	Yes	Yes	Yes	Yes	Yes	Yes	Yes
Control Variables	No	No	Yes	Yes	Yes	Yes	Yes	Yes
Number of Observations	4,175	4,175	4,175	3,820	4,146	4,175	4,175	4,175
Number of Districts	261	261	261	251	261	261	261	261
*R* ^*2*^ (within)	.56	.56	.58	.40	.21	.13	.63	.77

*Notes*: Control variables are lagged by one year and include rural population share, age composition of the female population, education shares, and log per capita expenditure. Robust standard errors are shown in brackets and are clustered at the district level. Full results are reported in Online Resource 1, Tables S3 and S4.

*Source*: SUSENAS household surveys.

^†^ *p* < .10; *p < .05; **p < .01

All coefficients associated with the control variables have the expected signs.^3^ Higher urbanization and higher education are both associated with lower fertility.^4^ We find a positive income effect, which may seem counterintuitive but is in line with theoretical predictions: as long as children are considered a normal good, the pure income effect should be positive if urbanization, education, hoarding, and price effects (particularly the opportunity cost of time) are controlled for. The statistically significant positive income effect is also observed for rural areas but diminishes and loses precision for urban areas. This supports the view that children have a different value in urban areas than in rural ones, where relatively wealthier parents tend to have fewer but better-educated children, possibly because the return to education may exceed the return to child labor.

Columns 6–8 in Table 2 present estimates of the effects of electrification by age group, showing that the effect of electricity is always statistically significant and negative. The order of magnitude for the age group 25–34 is comparable with that for the overall female population of reproductive age; however, for older and younger age groups, the effects appear smaller.

Although the effect of electricity coverage on fertility is quite robust across these different specifications, it cannot be excluded that time-varying district-specific shocks or confounding unobserved economic development still lead to an omitted variables bias. To assess this potential bias, we add time-varying village characteristics from the village census (PODES) to the set of control variables. A summary of the results is presented in Table 3. These PODES variables are all listed in Table 1 and primarily relate to the availability of infrastructure, including water and education. Because PODES data are not available in all years, the benchmark specification taken from column 3 in Table 2 is reestimated on the reduced sample of observations, yielding a coefficient of –0.19 (Table 3, column 1). The estimates do not seem sensitive to adding the PODES characteristics: the electrification coefficient decreases only slightly to –0.18 (Table 3, column 2). To place these results in perspective, when we compare the estimated coefficients with the total change in the average number of live births over the observation period of 16.5 %, electricity does in fact explain a large part of it. The reduced-form point estimate in column 2 implies that over the period 1993–2010, electrification alone reduced the number of live births per woman by 0.067 (i.e., 0.1799 × 0.3731), corresponding to about 19 % (i.e., 0.067 / 0.350) of the total change in fertility over the same period.^5^ This effect seems sizable, especially compared with factors that have traditionally been credited for decreasing fertility, such as increased female education (see, e.g., Molyneaux and Gertler 2000), for which we estimate the contribution to be about 27 %. The effect, though, is still a bit lower than what was attributed to electrification in Brazil (Potter et al. 2002).

**Table 3:** Robustness of impact of electrification on fertility to including village economic and infrastructure variables, women aged 15–49, PODES years only

	(1)	(2)
Electricity Coverage	–0.1941**	–0.1799**
	[0.0646]	[0.0661]
Year Dummy Variables	Yes	Yes
District Fixed Effects	Yes	Yes
Control Variables	Yes	Yes
PODES Variables	No	Yes
Number of Observations	1,304	1,296
Number of Districts	261	261
*R* ^*2*^ (within)	.50	.52

*Notes*: PODES years are 1996, 2000, 2003, 2006, and 2008. Control variables are lagged by one year and include rural population share, age composition of the female population, education shares, and log per capita expenditure. Robust standard errors are shown in brackets and are clustered at the district level. Full results are reported in Online Resource 1, Table S6.

*Source*: SUSENAS household surveys and PODES village census.

**p < .01

These results are robust to the specification and choice of control variables, even though some of the PODES variables do appear statistically significant. The results are also not dependent on the inclusion or exclusion of certain subsets of PODES variables, which could be the case if the PODES variables were highly colinear.^6^ Finally, the effect also holds if, instead of the district electrification rate, the district-average of village electrification is used as explanatory variable, where village electrification is defined as at least one household in the village reporting the use of electricity as the main source of lighting. This should further reduce a possible endogeneity problem because the focus is then more on potential access than on actual access.^7^ Obviously, with this specification, we lose some variation at the lower end, thus reducing the estimated coefficient; but at least for the entire sample, the effect is still significantly negative. Overall, this robustness suggests that it is unlikely that the estimated effect of electrification on fertility is driven by unobserved economic development or modernity and therefore should approximate the true causal effect. If these unobservable characteristics accounted for part of the estimated effect of electrification, we would expect this estimate to be sensitive. Nevertheless, in the remainder of the analysis, we stick to the conservative estimates and include the PODES variables where possible. Among the PODES characteristics that can be associated with access to education and with modernity (such as the existence of a shopping complex), we find a negative sign; public health facilities for maternity care have a positive effect on fertility.

To further document the robustness of our findings, we conduct a few more sensitivity checks. First, we reestimate the model on either only those districts where in 1993 the electrification rate was below 50 % (roughly the median) or above 50 % to see whether the initial electrification level matters.^8^ The estimated effects are very similar to the corresponding estimates shown in Table 2 and 3.

Finally, we reestimate the model controlling for the presence of a power plant in the same *or* neighboring district and alternatively using the presence of a power plant in the same *and* a neighboring district as an instrument for the electrification rate. The results are presented in Table 4. If we control for only the presence of power plants (column 2), the electricity effect is not different from the previous estimates (reproduced in column 1). This is also the case if we consider only those power plants that have been in operation for at least five years (column 3). If we use the IVs, the electricity effect remains negative and statistically significant but increases in its absolute size, especially if we use all power plants as the instrument. This could indicate a weak instrument problem, although at least in column 4, the *F* statistics suggest that the instruments are strong. The results for our preferred instrument—namely, power plants that are older than five years—are closer to the results in Table 3, yet still somewhat larger. The difference in size is consistent with the interpretation of the LATE, given that the explanatory power of the instrument is likely to be driven largely by less remote or urban areas that have better access to local power plants. Similarly, Dinkelman (2011) and Lipscom et al. (2013) also found larger effects from IV strategies compared with noninstrumented estimates. The over-identifying restrictions tests do not reject the validity of the IVs. Hence, although we are inclined to interpret the more conservative fixed-effects estimates as preferred results, the IV estimates underline the robustness of our findings.

**Table 4:** Impact of electrification on fertility (average number of live births), women aged 15–49, 1993–2010, IV estimates

		Power Plants as Control Variables		Power Plants as IVs	
	Table 2 Results	All Plants	>5 Years	All Plants	>5 Years
Electricity Coverage	–0.2374**	–0.2288**	–0.2346**	–0.4789**	–0.3788*
	[0.0414]	[0.0419]	[0.0412]	[0.1316]	[0.1742]
Power Plants District		–0.0021	0.0056		
		[0.0248]	[0.0242]		
Power Plants Neighbor		–0.0247	–0.0130		
		[0.0174]	[0.0195]		
First-Stage IV					
Power plants district				0.0332**	0.0404**
				[0.0106]	[0.0105]
Power plants neighbor				0.0892**	0.0567**
				[0.0086]	[0.0076]
Joint Significance Instruments					
*F* statistic (clustered standard errors)				14.6	6.9
*F* statistic (nonclustered standard errors)				69.3	38.3
Over-Identifying Restrictions Test					
χ^2^(1) test statistic				0.01	0.08
*p* value				.90	.78
Year Dummy Variables	Yes	Yes	Yes	Yes	Yes
District Fixed Effects	Yes	Yes	Yes	Yes	Yes
SUSENAS Control Variables	Yes	Yes	Yes	Yes	Yes
Number of Observations	4,175	4,175	4,175	4,175	4,175
Number of Districts	261	261	261	261	261
*R* ^*2*^ (within)	.58	.58	.58	.57	.57

*Notes:* Control variables are lagged by one year and include rural population share, age composition of the female population, education shares, and log per capita expenditure. Standard errors are shown in brackets.

*Source*: SUSENAS household surveys.

*p < .05; **p < .01

### How Does Electrification Affect Fertility? Understanding the Transmission Channels

Guided by our theoretical framework, we now explore in more detail the channels through which electricity may affect fertility.

#### Does Electrification Affect Fertility Through Changes in Children’s and Women’s Labor Market Participation?

As we noted earlier, access to electricity may influence the shadow price of children by affecting both the direct and indirect cost of raising children. Table 5 shows that child labor indeed decreases with electricity coverage. In a district that is electrified, the share of children working decreases on average by 6 percentage points. If child labor is introduced in the fertility equation, the coefficient of child labor is not statistically significant. Overall, this suggests that electrification substitutes for child labor, which is in itself an interesting finding, but changes in child labor do not seem to cause changes in fertility.

**Table 5:** Impact of electrification on fertility, and the role of child and female labor, 1993–2010, PODES years only

	Child Works	Female Works	Fertility (age 15–49)
	(1)	(2)	(3)
Electricity Coverage	–0.0637**	–0.0698*	–0.1860**
	[0.0244]	[0.0294]	[0.0687]
Child Works (ages 10–15)			0.1299
			[0.1219]
Female Works			–0.0231
			[0.0803]
Male Works			–0.3085^†^
			[0.1834]
Year Dummy Variables	Yes	Yes	Yes
District Fixed Effects	Yes	Yes	Yes
Control Variables	Yes	Yes	Yes
PODES Variables	Yes	Yes	Yes
Number of Observations	1,296	1,296	1,296
Number of Districts	261	261	261
*R* ^*2*^ (within)	.47	.46	.52

*Notes*: PODES years are 1996, 2000, 2003, 2006, and 2008. Control variables are lagged by one year and include rural population share, age composition of the female population, education shares, and log per capita expenditure. Robust standard errors are shown in brackets and are clustered at the district level. Full results are reported in Online Resource 1, Table S10.

*Source*: SUSENAS household surveys.

^†^ *p* < .10; *p < .05; **p < .01

Electricity coverage also lowers female labor market participation by roughly the same extent that it lowers child labor. This might seem counterintuitive because the availability of electricity may open up new labor market opportunities. Similar to child labor, we find no effects of female labor participation on fertility. We also control for the district-specific share of men working to account for the complete allocation of time within the household. The share of men working is associated with fertility decline, but these effects are difficult to interpret because they account solely for time allocation and do not capture wage or income effects. The absence of an effect of electrification on female labor market participation is in contrast with the findings by Dinkelman (2011) for South Africa, who found a significant positive effect of household electrification on female employment and argued that this increase stems from a release of women from home production and from the creation of new micro-enterprises. For India, Van de Walle et al. (2013) found only moderate effects, observing an increase in female casual work but not regular wage work.

#### Does Electrification Affect Fertility Through Increased Exposure to Modern Media?

Media exposure is another possible channel linking electricity and fertility given that it may change the fertility preferences through the promotion of specific role models or by providing information on modern contraception. This exposure may reduce fertility through a more efficient prevention of unwanted births by reducing the price of not having a child and conversely increasing the relative price of having a child.

The results in Table 6, columns 1 and 2, suggest that electricity coverage increases exposure to TV while simultaneously decreasing the exposure to newspapers, suggesting that the former is a substitute to the latter.^9^ The results shown in column 3 suggest that exposure to TV increases the use of modern contraception by about 12 percentage points. There is no effect on the use of traditional contraception, such as withdrawal. This result is what we would expect because the government in Indonesia uses television to promote modern family planning and discourages traditional methods (Dewi et al. 2013).

**Table 6:** Impact of electrification on fertility, and the role of media, 1993–1998

			Contraception Used				
	TV	Newspaper	All	Traditional	Fertility (ages 15–49)		
	(1)	(2)	(3)	(4)	(5)	(6)	(7)
Electricity Coverage	0.1848**	–0.0518*	0.0161	–0.0039		–0.1030^†^	–0.1227*
	[0.0400]	[0.0222]	[0.0187]	[0.0030]		[0.0578]	[0.0597]
Watch TV			0.1176**	0.0033	–0.0948*	–0.0739^†^	
			[0.0150]	[0.0021]	[0.0455]	[0.0440]	
Listen to Radio			–0.0165	–0.0013	0.0914*	0.0878	
			[0.0143]	[0.0027]	[0.0582]	[0.0571]	
Read Newspaper			–0.1496**	–0.0013	–0.0868	–0.0964	
			[0.0273]	[0.0037]	[0.0833]	[0.0831]	
Contraceptives Used					–0.1031	–0.1042	–0.1183
					[0.0894]	[0.0888]	[0.0877]
Traditional Contraceptives Used					0.0126	–0.0268	–0.0710
					[0.5689]	[0.5702]	[0.5765]
Year Dummy Variables	Yes	Yes	Yes	Yes	Yes	Yes	Yes
District Fixed Effects	Yes	Yes	Yes	Yes	Yes	Yes	Yes
Control Variables	Yes	Yes	Yes	Yes	Yes	Yes	Yes
PODES Variables	No	No	No	No	No	No	No
Number of Observations	1,305	1,305	1,305	1,305	1,566	1,566	1,566
Number of Districts	261	261	261	261	261	261	261
*R* ^*2*^ (within)	.91	.64	.78	.09	.41	.41	.41

*Notes*: Control variables are lagged by one year and include rural population share, age composition of the female population, education shares, and log per capita expenditure. Robust standard errors are shown in brackets and are clustered at the district level. Full results are reported in Online Resource 1, Table S11.

*Source*: SUSENAS household surveys.

^†^ *p* < .10; *p < .05; **p < .01

TV exposure enters negatively and statistically significant in the fertility equation. When it is introduced together with electricity, the effect of TV exposure is still statistically significant, but the effect of electricity is slightly lower and less precise than a regression in which TV exposure is excluded. Thus, TV exposure indeed seems to be a relevant transmission channel linking electricity and fertility. The point estimate implies that an increase of the share of women exposed to TV by 1 standard deviation (SD) (i.e., by 43 %) reduces the average number of live births per woman by 0.022 (i.e., by about 1.2 %). This means that the TV effect explains about one-quarter of the total reduction in fertility that can be attributed to the increase in electrification. Our point estimate is comparable to the effect identified by La Ferrara et al. (2012). For Brazil, they found that the exposure to the signal of cable TV showing soap operas reduced the average number of live births per woman by 0.027, with an only slightly higher overall mean in their dependent variable.^10^

To further assess the effects on desired fertility, we turn to the DHS data. Again, we need to be cautious when interpreting the results based on the DHS, but these data can still provide interesting insights. Moreover, when replicating the analysis of electrification on fertility, we find results that are qualitatively similar to those based on the district panel.^11^ The average desired number of children decreases over time, suggesting that next to the use of family planning devices, and hence a reduction in unwanted births, a reduction in the number of wanted births is a major driver of Indonesia’s fertility decline. The preferred number of children increases with age, mainly reflecting cohort effects, but also the fact that respondents *ex post* tend to rationalize actual fertility: that is, older women may overreport the children that they would have preferred at the beginning of their fertility cycle (BPS 2008). We apply a Poisson regression with the number of desired children as dependent variable and dummy variables indicating the availability of electricity and whether the household has a TV set as main explanatory variables. The control variables include age, a dummy variable for living in a rural area, education, asset ownership, and DHS wave indicators.

Column 1 of Table 7 presents the reduced form showing that electricity is negatively correlated with desired fertility. Women in households with electricity report an ideal number of children that is lower by roughly 5 % or 0.15 children (marginal effect computed at the sample mean) compared with the reported ideal number by women in households without electricity. When TV is also introduced (column 2), both variables have a negative effect. The effect associated with TV exposure implies a reduction in the number of desired children by about 2.5 %, possibly through new information, new role models, and so forth. The effect associated with electricity is roughly unchanged. The upshot from these regressions is that electricity has an effect on actual and desired fertility that is independent of TV exposure.

**Table 7:** Impact of electrification on fertility preferences and the role of media, 1991–2007, DHS data, Poisson model

	Number of Desired Children	
	(1)	(2)
Electricity Coverage	–0.047**	–0.053**
	[0.005]	[0.005]
Watch TV		–0.024**
		[0.005]
DHS Wave	Yes	Yes
Control Variables	Yes	Yes
Number of Observations	107,137	107,137

*Notes*: Control variables include age, education, an asset index, and a dummy variable for rural areas. Robust standard errors (i.e., corrected for intracluster correlation) are shown in brackets. Full results are reported in Online Resource 1, Table S13.

*Source*: DHS, various years.

**p < .01

#### Does Electrification Affect Fertility Through Reduced Child Mortality?

Finally, we examine whether electrification also affects child mortality—for example, by improved quality of available health care, including better conditions of maternal care and attended births. We also examine whether child mortality affects actual and desired fertility. If risk-averse parents who aim for a certain number of children that survive until adulthood expect higher survival chances for their offspring, they may reduce the number of desired and actual pregnancies. The estimation in column 1 in Table 8 shows that electrification per se, as expected, significantly reduces child mortality. Furthermore, child mortality—here approximated by the average mortality in a woman’s cluster^12^—increases actual fertility (column 2): that is, parents seem to anticipate that some of their children may die. When cluster-specific mortality is introduced, the electricity effect decreases, suggesting that the electricity effect passes at least partly through reduced mortality.^13^ The same holds for desired fertility (comparing column 3 in Table 8 with column 1 in Table 7).

**Table 8:** Impact of electrification on fertility and fertility preferences, and the role of child mortality, 1991–2007, DHS data, Poisson model

	Mortality	Fertility	Desired Fertility
	(1)	(2)	(3)
Electricity Coverage	–0.089**	–0.008	–0.031**
	[0.009]	[0.006]	[0.004]
Average Cluster Mortality		0.456**	0.393**
		[0.005]	[0.006]
DHS Wave	Yes	Yes	Yes
Control Variables	Yes	Yes	Yes
Number of Observations	131,409	131,409	107,137

*Notes*: Control variables include age, education, an asset index, and a dummy variable for rural areas. Robust standard errors (i.e., corrected for intracluster correlation) are shown in brackets. Full results are reported in Online Resource 1, Table S14.

*Source*: DHS, various years.

**p < .01

## Conclusion

Theoretically, the availability and use of electricity may affect fertility via multiple pathways. This article empirically examines several of these pathways. The reduced-form estimates suggest that expanding electrification in Indonesia explains about 19 % to 25 % of the overall decline in the fertility rate between 1993 and 2010, depending on the specification. This seems a sizable effect, especially if we compare this with factors that have traditionally been credited for decreasing fertility, such as increased female education, for which we estimate the contribution to be about 27 %. The estimate is robust to a large number of robustness checks, including an IV estimator.

We also made an attempt to unpack the net effect to further understand how electricity affects fertility. Although the availability of electricity does seem to reduce women’s and children’s labor market participation, it does not affect fertility through this channel. However, we find evidence that the availability of electricity increases exposure to TV, and by this channel reduces fertility. The point estimate implies that an increase of the share of women exposed to TV by 1 standard deviation, (i.e., 43 %) reduces the average number of live births per woman by 0.022 (i.e., by about 1.2 %). TV explains about one-quarter of the total fertility effect. Further analysis suggests that the effect through TV is partly explained by changes in desired fertility and a more effective use of modern contraception. However, assuming that watching TV simply reduces the time spent on physical intimacy is also plausible. Anecdotic evidence seems to suggest that this effect is important not only in Indonesia but, for example, also in India or China (Dewi et al. 2013; Johnson 2001). However, because we cannot test this hypothesis formally, it remains speculation. Finally, we find that the availability of electricity seems to reduce child mortality and, by this channel, also fertility. However, given that the latter results are based on pooled cross-sectional DHS data, these results are more vulnerable to omitted variable bias and require caution to be interpreted as causal effects. Nevertheless, these results are informative and consistent with the other results. More generally, although we cannot fully rule out some remaining bias due to time-varying district-specific unobservable factors that are correlated with both electrification and fertility decisions, we see the strength of our quasi-experimental approach to be the potentially high degree of external validity because our analysis is based on a very large and representative sample covering a period of almost two decades. Although there may still be limitations to using our results to provide projections for other countries, our findings suggest that the fertility-reducing effects from electrification should be taken in to account when the costs of electricity rollout are compared with its benefits. This is potentially important for many countries in Sub-Saharan Africa where fertility is still very high and grid electricity, at least in rural areas, is still rare.

## Electronic supplementary material

Online Resource 1 (DOCX 108 kb)

## Footnotes

1 District splits followed almost entirely subdistrict boundaries within the relevant district and did not affect borders with neighboring districts. See Fitrani et al. (2005) for a more complete account of this process. Statistics Indonesia maintains a full list of district codes over time (see http://www.bps.go.id/mstkab/mfkab_03_09.pdf).

2 This figure shows the decline in the fertility rate that includes both a general decline in fertility and changes in the age composition over time.

3 The coefficients for the control variables are not shown here, but the complete estimation results are reported in Online Resource 1.

4 We also considered controlling for contraceptive use and the average age of marriage for the relevant age reference group of women given that the strong decline in the fertility rate has been associated with the rise in age of marriage and use of modern contraceptives. The estimates appear to be robust to including these variables because it does not affect the electrification coefficients. The effect of the share of women using modern contraceptives is negative and highly significant, but the use of traditional contraceptives does not have any effect. As we examine in more detail later, the use of modern contraception is a possible pathway for the effect of electrification on fertility; hence, this variable may capture parts of the electricity effect. We therefore leave contraceptive use out of the main specification and treat it solely as transmission channel. The age-at-marriage variable shows a statistically significant negative coefficient, confirming that an increase in the average age at first marriage is associated with a reduction in the average number of live births per woman. However, we also suspect that this variable is potentially endogenous given that the onset of fertility is correlated with total fertility and the age at marriage. We therefore also omit age at marriage from our preferred specification. The results including these variables are presented in Online Resource 1, Table S5.

5 The average electrification rate increased by 37.31 percentage points from 57.6 % in 1993 to 94.9 % in 2010, and the average number of live births per woman declined by 0.35 from 2.118 live births in 1993 to 1.768 in 2010.

6 This is shown in Online Resource 1, Table S7.

7 These results are shown in Online Resource 1, Table S8.

8 These results are shown in Online Resource 1, Table S9.

9 Because the media variables are available in the SUSENAS surveys only from 1993 to 1998, the number of observations is smaller than those reported in Table 5. For this period, we have PODES data only for 1996; therefore, PODES variables are not included as controls in the fixed-effects regressions.

10 Jensen and Oster (2009) estimated for India that exposure to cable TV reduced the probability of a woman being pregnant at the time of the survey by 4 % to 7 %.

11 To further check the robustness of our results, we reestimated Eq. (1) with the five rounds of pooled DHS data (1991, 1994, 1997, 2002/2003, and 2007). These estimates provide results consistent with those obtained through the district panel. The full results are reported in Online Resource 1 (Table S12).

12 This average is computed without taking into account the experienced child mortality of the mother under study. In the Indonesian DHS, a cluster contains on average 60 eligible women. The average cluster sample size is relatively small for calculating mortality rates, which may reduce the precision of the estimates. Unfortunately, we do not have access to census and vital statistics.

13 Compare column 2 in Table 8 with column 2 in Table S12 in Online Resource 1.
