# Supplementary material for: Does Electrification Spur the Fertility Transition? Evidence From Indonesia
Source: Demography. 2015 Aug 26;52(5):1773–96. doi: 10.1007/s13524-015-0420-3 (PMC4605992; doi:10.1007/s13524-015-0420-3)
Supplement: Supplementary file 1 — (DOCX 108 kb) [file 13524_2015_420_MOESM1_ESM.docx]

**Online Resource 1**

**Does Electrification Spur the Fertility Transition? Evidence From Indonesia**

Michael Grimm^1,2,3^, Robert Sparrow^3,4^, Luca Tasciotti^2^

Michael Grimm

e-mail: michael.grimm@uni-passau.de

^1^ Department of Economics, University of Passau, Innstraße 29, 94032 Passau, Germany

^2^ Erasmus University Rotterdam, P.O. Box 29776, 2502 LT The Hague, The Netherlands

^3^ IZA, Bonn, Germany

^4^ Arndt-Corden Department of Economics, Crawford School of Public Policy, Australian National University, Canberra, ACT 0200, Australia

Table S1 Used data sources

| Name of data source | Observation unit (raw data) | Unit of analysis | N | Years |
| --- | --- | --- | --- | --- |
| Susenas | Households and women 15 to 49 years living in these households | Districts | 261 districts  (260 in 2006) | 1993 to 2010 (annually) |
| Podes | Rural villages and urban precincts | Districts | 261 districts  (260 in 2006) | 1996, 2000, 2003, 2006, 2008 |
| Demographic and Health Survey (DHS) | Women 15 to 49 years | Women 15 to 49 years | 131,409 (pooled) | 1991, 19994, 1997, 2002/03, 2007 |
| Power plants | Power plant | Power plants | 133 | 1993-2010 (annually) |

*Source:* Own representation.

Table S2 Synthesis of results

|  | Demand | | Supply | Actual fertility |
| --- | --- | --- | --- | --- |
|  | Desired fertility ^b)^ | Use of modern contraception | Survival chances ^b)^ |  |
| Electricity | --- |  | +++ | -- |
|  |  |  |  |  |
| Transmission channels (effect of electricity through)^a)^ |  |  |  |  |
| Children’s labour supply (--) |  |  |  | n.s. |
| Women’s labour supply (--) |  |  |  | n.s. |
| TV exposure (++) | --- | ++ |  | - |
| Child mortality ^b)^ (---) | +++ |  |  | +++ |

*Note:* ^a)^ In parentheses direction of effect of electricity on corresponding channel variable. “-“ (“+“) negative (positive) effect significant at 10%, “--“ (“++“) negative (positive) effect significant at 5%, “---“(“+++“) negative (positive) effect significant at 1%. n.s. stands for “not significant”. ^b)^ These channels have been analysed with the DHS data and hence cannot necessarily be interpreted as causal. Channels with empty fields have not been analysed.

*Source:* Own representation.

Table S3 Impact of electrification on fertility (average number of live births), women ages 15-49, all *lagged* explanatory variables, 1993-2010

|  | Random effects | Fixed effects | | | | |
| --- | --- | --- | --- | --- | --- | --- |
|  | All HH | All HH | | | Rural HH | Urban HH |
|  | (1) | (2) | (3) | (4) | (5) | (6) |
| Electricity coverage | -0. 3227** | -0.2695** | -0.2140** | -0.2374** | -0.1718** | -0.1826* |
|  | [0.0390] | [0.0395] | [0.0416] | [0.0414] | [0.0497] | [0.0851] |
| Rural population share |  |  | 0.1661** | 0.1812** |  |  |
|  |  |  | [0.0562] | [0.0550] |  |  |
| Composition of female population (ref: share aged 15-19) |  |  |  |  |  |  |
| Share aged 20-24 |  |  | -0.3183* | -0.3010* | 0.0612 | 0.3382* |
|  |  |  | [0.1530] | [0.1526] | [0.3027] | [0.1553] |
| Share aged 25-29 |  |  | -0.1966 | -0.1804 | -0.1224 | 0.2991* |
|  |  |  | [0.1398] | [0.1424] | [0.2787] | [0.1338] |
| Share aged 30-34 |  |  | 0.1602 | 0.1309 | 0.2841 | 0.1954 |
|  |  |  | [0.1625] | [0.1608] | [0.2022] | [0.1459] |
| Share aged 35-39 |  |  | 0.3716+ | 0.3359+ | 0.2599 | 0.3368+ |
|  |  |  | [0.1970] | [0.1939] | [0.2836] | [0.1871] |
| Share aged 40-44 |  |  | 0.1620 | 0.1146 | -0.2015 | 0.3532+ |
|  |  |  | [0.2039] | [0.1991] | [0.3631] | [0.1902] |
| Share aged 45-49 |  |  | 0.1239 | 0.1448 | -0.1239 | 0.2954 |
|  |  |  | [0.2013] | [0.1967] | [0.2936] | [0.1943] |
| Highest education completed by women aged 15-49 (ref: none) |  |  |  |  |  |  |
| Primary |  |  | -0.3593** | -0.3546** | -0.2048 | -0.2216* |
|  |  |  | [0.0814] | [0.0797] | [0.1272] | [0.0859] |
| Junior secondary |  |  | -0.3381** | -0.4001** | -0.3600* | -0.2368* |
|  |  |  | [0.0802] | [0.0832] | [0.1435] | [0.0979] |
| Senior secondary |  |  | -0.4447** | -0.5114** | -0.3433* | -0.2306** |
|  |  |  | [0.0924] | [0.0957] | [0.1539] | [0.0795] |
| Higher |  |  | -0.3902** | -0.3769** | -0.1268 | -0.0324 |
|  |  |  | [0.1425] | [0.1409] | [0.2327] | [0.1330] |
| Log per capita expenditure |  |  |  | 0.1092** | 0.1229** | 0.0199 |
|  |  |  |  | [0.0214] | [0.0386] | [0.0249] |
| Constant | 2.2693** | 2.0521** | 2.1419** | 0.6512* | 1.0580* | 1.7444** |
|  | [0.0307] | [0.0354] | [0.1422] | [0.3006] | [0.4642] | [0.2639] |
| Year dummy variables | Yes | Yes | Yes | Yes | Yes | Yes |
| Number of observations | 4175 | 4175 | 4175 | 4175 | 3820 | 4146 |
| Number of districts | 261 | 261 | 261 | 261 | 251 | 261 |
| R^2^ (within) | 0.56 | 0.56 | 0.57 | 0.58 | 0.40 | 0.21 |

*Note*: Robust standard errors are shown in brackets and are clustered at the district level.

Source: SUSENAS household surveys.

**^†^***p* < .10; **p* < .05; ***p* < .01

Table S4 Impact of electrification on fertility (average number of live births) by age group, all *lagged* explanatory variables, 1993-2010, fixed effects regressions

|  | 15 - 24 | 25 - 34 | 35 - 49 |
| --- | --- | --- | --- |
|  | (1) | (2) | (3) |
| Electricity coverage | -0.0692** | -0.2170** | -0.1484+ |
|  | [0.0171] | [0.0603] | [0.0811] |
| Rural population share | 0.0768** | 0.2139* | 0.3327** |
|  | [0.0213] | [0.0857] | [0.1075] |
| Composition of female population (ref: share aged 15-19) |  |  |  |
| Share aged 20-24 | 0.1549* | -0.7278** | -0.7952** |
|  | [0.0745] | [0.2184] | [0.3042] |
| Share aged 25-29 | 0.3320** | -0.2966 | -1.3286** |
|  | [0.0827] | [0.2016] | [0.2782] |
| Share aged 30-34 | 0.1199 | 0.0479 | -1.0191** |
|  | [0.0813] | [0.2163] | [0.3203] |
| Share aged 35-39 | 0.0839 | 0.0937 | -0.7852* |
|  | [0.0899] | [0.2483] | [0.3700] |
| Share aged 40-44 | 0.0421 | -0.2416 | -1.2447** |
|  | [0.0981] | [0.2640] | [0.3940] |
| Share aged 45-49 | 0.0598 | -0.4335+ | -0.7647+ |
|  | [0.0982] | [0.2617] | [0.4387] |
| Highest education completed by women aged 15-49 (ref: none) |  |  |  |
| Primary | -0.1089** | -0.3720** | -0.5050** |
|  | [0.0383] | [0.1038] | [0.1556] |
| Junior secondary | -0.0620 | -0.5987** | -0.5069** |
|  | [0.0435] | [0.1045] | [0.1559] |
| Senior secondary | -0.0813* | -0.6722** | -0.6803** |
|  | [0.0410] | [0.1367] | [0.1857] |
| Higher | 0.0727 | -0.1918 | -1.2327** |
|  | [0.0651] | [0.1973] | [0.2670] |
| Log per capita expenditure | 0.0324** | 0.1313** | 0.1730** |
|  | [0.0112] | [0.0289] | [0.0403] |
| Constant | -0.2009 | 0.4567 | 1.9285** |
|  | [0.1705] | [0.4035] | [0.5897] |
| Year dummy variables | Yes | Yes | Yes |
| Number of observations | 4175 | 4175 | 4175 |
| Number of districts | 261 | 261 | 261 |
| R^2^ (within) | 0.13 | 0.63 | 0.77 |

*Note*: Robust standard errors are shown in brackets and are clustered at the district level.

Source: SUSENAS household surveys.

**^†^***p* < .10; **p* < .05; ***p* < .01

Table S5 Sensitivity of the results to the inclusion of contraceptive use and first age of marriage

|  | (1) | (2) | (3) |
| --- | --- | --- | --- |
| Electricity coverage | -0.2374** | -0.2245** | -0.2430** |
|  | [0.0414] | [0.0409] | [0.0408] |
| Rural population share | 0.1812** | 0.1806** | 0.1665** |
|  | [0.0550] | [0.0547] | [0.0545] |
| Composition of female population (ref: share aged 15-19) |  |  |  |
| Share aged 20-24 | -0.3010* | -0.2697+ | -0.2797+ |
|  | [0.1526] | [0.1517] | [0.1536] |
| Share aged 25-29 | -0.1804 | -0.1005 | -0.1326 |
|  | [0.1424] | [0.1431] | [0.1419] |
| Share aged 30-34 | 0.1309 | 0.2137 | 0.2159 |
|  | [0.1608] | [0.1641] | [0.1573] |
| Share aged 35-39 | 0.3359+ | 0.4176* | 0.4420* |
|  | [0.1939] | [0.1956] | [0.1909] |
| Share aged 40-44 | 0.1146 | 0.1854 | 0.2735 |
|  | [0.1991] | [0.1976] | [0.1952] |
| Share aged 45-49 | 0.1448 | 0.1821 | 0.3006 |
|  | [0.1967] | [0.1969] | [0.1888] |
| Highest education completed by women aged 15-49 (ref: none) |  |  |  |
| Primary | -0.3546** | -0.3360** | -0.3080** |
|  | [0.0797] | [0.0798] | [0.0803] |
| Junior secondary | -0.4001** | -0.4003** | -0.2881** |
|  | [0.0832] | [0.0828] | [0.0873] |
| Senior secondary | -0.5114** | -0.5149** | -0.3958** |
|  | [0.0957] | [0.0948] | [0.0994] |
| Higher | -0.3769** | -0.4173** | -0.1370 |
|  | [0.1409] | [0.1433] | [0.1449] |
| Log per capita expenditure | 0.1092** | 0.1075** | 0.1036** |
|  | [0.0214] | [0.0210] | [0.0206] |
| Contraceptives used |  | -0.1716** |  |
|  |  | [0.0605] |  |
| Traditional contraceptives used |  | -0.1020 |  |
|  |  | [0.3804] |  |
| Average age of first marriage |  |  | -0.0297** |
|  |  |  | [0.0085] |
| Constant | 0.6512* | 0.6804* | 1.1868** |
|  | [0.3006] | [0.2965] | [0.3261] |
| Year dummy variables | Yes | Yes | Yes |
| Number of observations | 4175 | 4175 | 4175 |
| Number of districts | 261 | 261 | 261 |
| R^2^ (within) | 0.58 | 0.58 | 0.58 |

*Note*: Robust standard errors are shown in brackets and are clustered at the district level.

Source: SUSENAS household surveys.

**^†^***p* < .10; **p* < .05; ***p* < .01

Table S6 Robustness of impact of electrification on fertility to including village economic and infrastructure variables, all *lagged* explanatory variables, 1993-2010, women ages 15-49, fixed effects regressions

|  | All years | Podes years | |
| --- | --- | --- | --- |
|  | (1) | (2) | (3) |
| Electricity coverage | -0.2374** | -0.1941** | -0.1799** |
|  | [0.0414] | [0.0646] | [0.0661] |
| Rural population share | 0.1812** | 0.2999** | 0.2829** |
|  | [0.0550] | [0.0931] | [0.0893] |
| Composition of female population (ref: share aged 15-19) |  |  |  |
| Share aged 20-24 | -0.3010* | -0.4461+ | -0.4611+ |
|  | [0.1526] | [0.2470] | [0.2523] |
| Share aged 25-29 | -0.1804 | -0.1748 | -0.1889 |
|  | [0.1424] | [0.2878] | [0.2805] |
| Share aged 30-34 | 0.1309 | -0.0637 | -0.0775 |
|  | [0.1608] | [0.3029] | [0.2955] |
| Share aged 35-39 | 0.3359+ | 0.1983 | 0.1618 |
|  | [0.1939] | [0.3174] | [0.3120] |
| Share aged 40-44 | 0.1146 | 0.0643 | 0.1299 |
|  | [0.1991] | [0.3079] | [0.3140] |
| Share aged 45-49 | 0.1448 | 0.2262 | 0.2675 |
|  | [0.1967] | [0.3146] | [0.3225] |
| Highest education completed by women aged 15-49 (ref: none) |  |  |  |
| Primary | -0.3546** | -0.4754** | -0.4897** |
|  | [0.0797] | [0.1071] | [0.1094] |
| Junior secondary | -0.4001** | -0.2608* | -0.2131+ |
|  | [0.0832] | [0.1222] | [0.1185] |
| Senior secondary | -0.5114** | -0.4650** | -0.4752** |
|  | [0.0957] | [0.1571] | [0.1496] |
| Higher | -0.3769** | -0.5515** | -0.5056* |
|  | [0.1409] | [0.1984] | [0.1982] |
| Log per capita expenditure | 0.1092** | 0.0702* | 0.0625+ |
|  | [0.0214] | [0.0353] | [0.0346] |
| Villages Characteristics (Podes) |  |  |  |
| Agriculture main activity |  |  | 0.0628 |
|  |  |  | [0.0568] |
| Market with (semi-)permanent building |  |  | 0.1171** |
|  |  |  | [0.0437] |
| Shopping complex |  |  | -0.0848+ |
|  |  |  | [0.0485] |
| Majority of traffic on asphalt road |  |  | -0.0063 |
|  |  |  | [0.0366] |
| Drinking water piped/pump |  |  | 0.0129 |
|  |  |  | [0.0434] |
| Nr. of primary schools |  |  | -0.0131* |
|  |  |  | [0.0055] |
| Nr. of junior secondary schools |  |  | 0.0272 |
|  |  |  | [0.0198] |
| Nr. of senior secondary schools |  |  | -0.0039 |
|  |  |  | [0.0182] |
| Nr. of maternity clinics/hospitals |  |  | 0.0478 |
|  |  |  | [0.0381] |
| Nr. of village health centers |  |  | -0.0534 |
|  |  |  | [0.0597] |
| Nr. of village maternity posts |  |  | 0.0385** |
|  |  |  | [0.0148] |
| Constant | 0.6512* | 1.1872* | 1.2482* |
|  | [0.3006] | [0.5192] | [0.5164] |
| Year dummy variables | Yes | Yes | Yes |
| Number of observations | 4175 | 1296 | 1296 |
| Number of districts | 261 | 261 | 261 |
| R^2^ (within) | 0.58 | 0.50 | 0.51 |

*Note*: Robust standard errors are shown in brackets and are clustered at the district level. Podes years are 1996, 2000, 2003, 2006 and 2008.

Source: SUSENAS household surveys and Podes village census.

**^†^***p* < .10; **p* < .05; ***p* < .01

Table S7 Sensitivity of the results with respect to the inclusion and exclusion of Podes controls

|  | (1) | (2) | (3) | (4) |
| --- | --- | --- | --- | --- |
| Electrification rate | -0.1799** | -0.2015** | -0.1755** | -0.1732** |
|  | [0.0661] | [0.0634] | [0.0658] | [0.0647] |
| Villages Characteristics (Podes) |  |  |  |  |
| Agriculture main activity | 0.0628 | 0.0519 |  |  |
|  | [0.0568] | [0.0565] |  |  |
| Market with (semi-)permanent building | 0.1171** | 0.0798+ |  |  |
|  | [0.0437] | [0.0447] |  |  |
| Shopping complex | -0.0848+ | -0.0966* |  |  |
|  | [0.0485] | [0.0482] |  |  |
| Majority of traffic on asphalt road | -0.0063 | -0.0028 |  |  |
|  | [0.0366] | [0.0362] |  |  |
| Drinking water piped/pump | 0.0129 | 0.0177 |  |  |
|  | [0.0434] | [0.0437] |  |  |
| Nr. of primary schools | -0.0131* |  | -0.0111* |  |
|  | [0.0055] |  | [0.0054] |  |
| Nr. of junior secondary schools | 0.0272 |  | 0.0216 |  |
|  | [0.0198] |  | [0.0203] |  |
| Nr. of senior secondary schools | -0.0039 |  | -0.0027 |  |
|  | [0.0182] |  | [0.0182] |  |
| Nr. of maternity clinics/hospitals | 0.0478 |  |  | 0.0224 |
|  | [0.0381] |  |  | [0.0385] |
| Nr. of village health centers | -0.0534 |  |  | -0.0869 |
|  | [0.0597] |  |  | [0.0559] |
| Nr. of village maternity posts | 0.0385** |  |  | 0.0387** |
|  | [0.0148] |  |  | [0.0147] |
| Year dummy variables | Yes | Yes | Yes | Yes |
| District fixed effects | Yes | Yes | Yes | Yes |
| SUSENAS controls | Yes | Yes | Yes | Yes |
| Number of observations | 1296 | 1296 | 1296 | 1296 |
| Number of districts | 261 | 261 | 261 | 261 |
| R^2^ (within) | 0.51 | 0.51 | 0.50 | 0.51 |

*Note*: Robust standard errors are shown in brackets and are clustered at the district level. Podes years are 1996, 2000, 2003, 2006 and 2008.

Source: SUSENAS household surveys and Podes village census.

**^†^***p* < .10; **p* < .05; ***p* < .01

Table S8 Impact of electrification on fertility using village level connection (at least one sampled household in the village is connected) instead of household level connection

|  | (1) | (2) | (3) |
| --- | --- | --- | --- |
| Village electrification rate | -0.1033* | -0.0533 | -0.0350 |
|  | [0.0448] | [0.0757] | [0.0763] |
| Year dummy variables | Yes | Yes | Yes |
| District fixed effects | Yes | Yes | Yes |
| SUSENAS controls | Yes | Yes | Yes |
| PODES controls | No | No | Yes |
| Number of observations | 4175 | 1296 | 1296 |
| Number of districts | 261 | 261 | 261 |
| R^2^ (within) | 0.57 | 0.49 | 0.51 |

*Note*: Robust standard errors are shown in brackets and are clustered at the district level.

Source: SUSENAS household surveys.

**^†^***p* < .10; **p* < .05; ***p* < .01

Table S9 Impact of electrification on fertility by initial electrification rate

|  | (1) | (2) | (3) | (4) |
| --- | --- | --- | --- | --- |
|  | Elec 1993 <.5 | Elec 1993 >=.5 | Elec 1993 <.5 | Elec 1993 >=.5 |
| Electrification rate | -0.2197** | -0.2797** | -0.1505 | -0.1941 |
|  | [0.0646] | [0.0727] | [0.1023] | [0.1442] |
| Year dummies | Yes | Yes | Yes | Yes |
| District fixed effects | Yes | Yes | Yes | Yes |
| SUSENAS controls | Yes | Yes | Yes | Yes |
| PODES controls | No | No | Yes | Yes |
| Number of observations | 2064 | 2111 | 640 | 656 |
| Number of districts | 129 | 132 | 129 | 132 |
| R^2^ (within) | 0.57 | 0.60 | 0.54 | 0.52 |

*Note*: Robust standard errors are shown in brackets and are clustered at the district level. Podes years are 1996, 2000, 2003, 2006 and 2008.

Source: SUSENAS household surveys and Podes village census.

**^†^***p* < .10; **p* < .05; ***p* < .01

Table S10 Impact of electrification on fertility, and the role of child and female labour, 1993-2010, fixed effects regressions

|  | Child works | Female works | Fertility ^1^ |
| --- | --- | --- | --- |
|  | (1) | (2) | (3) |
| Electricity coverage | -0.0637** | -0.0698* | -0.1860** |
|  | [0.0244] | [0.0294] | [0.0687] |
| Child works (age 10-15) |  |  | 0.1299 |
|  |  |  | [0.1219] |
| Female works |  |  | -0.0231 |
|  |  |  | [0.0803] |
| Male works |  |  | -0.3085+ |
|  |  |  | [0.1834] |
| Rural population share | 0.0712* | 0.0452 | 0.2893** |
|  | [0.0309] | [0.0364] | [0.0902] |
| Composition of female population (ref: share aged 15-19) |  |  |  |
| Share aged 20-24 | 0.3715** | 0.3702** | -0.4679+ |
|  | [0.0817] | [0.1092] | [0.2513] |
| Share aged 25-29 | 0.2804** | 0.1796 | -0.1598 |
|  | [0.0848] | [0.1122] | [0.2786] |
| Share aged 30-34 | 0.1799* | 0.3282** | -0.0539 |
|  | [0.0843] | [0.1148] | [0.2935] |
| Share aged 35-39 | 0.1489 | 0.0974 | 0.1645 |
|  | [0.1124] | [0.1281] | [0.3108] |
| Share aged 40-44 | 0.0878 | 0.1935 | 0.1379 |
|  | [0.0913] | [0.1334] | [0.3161] |
| Share aged 45-49 | -0.0837 | 0.0122 | 0.2934 |
|  | [0.0996] | [0.1210] | [0.3213] |
| Highest education completed by women aged 15-49 (ref: none) |  |  |  |
| Primary | -0.2003** | -0.1435** | -0.4900** |
|  | [0.0471] | [0.0457] | [0.1120] |
| Junior secondary | -0.1817** | -0.2847** | -0.2458* |
|  | [0.0447] | [0.0591] | [0.1219] |
| Senior secondary | -0.1799** | -0.2219** | -0.5154** |
|  | [0.0502] | [0.0736] | [0.1541] |
| Higher | -0.0437 | 0.0775 | -0.4945* |
|  | [0.0728] | [0.1065] | [0.1954] |
| Log per cap. exp. (t-1) | -0.0123 | -0.0003 | 0.0642+ |
|  | [0.0092] | [0.0131] | [0.0347] |
| Villages Characteristics (Podes) |  |  |  |
| Agriculture main activity | -0.0156 | 0.0160 | 0.0665 |
|  | [0.0161] | [0.0246] | [0.0562] |
| Market with (semi-)permanent building | 0.0177 | 0.0220 | 0.1160** |
|  | [0.0162] | [0.0223] | [0.0437] |
| Shopping complex | -0.0120 | -0.0059 | -0.0827+ |
|  | [0.0141] | [0.0226] | [0.0484] |
| Majority of traffic on asphalt road | 0.0185 | 0.0051 | -0.0061 |
|  | [0.0147] | [0.0153] | [0.0365] |
| Drinking water piped/pump | 0.0118 | 0.0174 | 0.0140 |
|  | [0.0130] | [0.0162] | [0.0430] |
| Nr. of primary schools | -0.0021 | -0.0057* | -0.0126* |
|  | [0.0014] | [0.0025] | [0.0055] |
| Nr. of junior secondary schools | 0.0019 | 0.0134+ | 0.0279 |
|  | [0.0058] | [0.0081] | [0.0198] |
| Nr. of senior secondary schools | 0.0049 | 0.0122 | -0.0070 |
|  | [0.0063] | [0.0078] | [0.0183] |
| Nr. of maternity clinics/hospitals | -0.0039 | 0.0064 | 0.0474 |
|  | [0.0122] | [0.0176] | [0.0383] |
| Nr. of village health centers | 0.0020 | -0.0394+ | -0.0544 |
|  | [0.0171] | [0.0231] | [0.0595] |
| Nr. of village maternity posts | -0.0243** | -0.0154* | 0.0391** |
|  | [0.0047] | [0.0066] | [0.0151] |
| Constant | 0.2458+ | 0.5375** | 1.4910** |
|  | [0.1293] | [0.2025] | [0.5633] |
| Year dummy variables | Yes | Yes | Yes |
| Number of observations | 1296 | 1296 | 1296 |
| Number of districts | 261 | 261 | 261 |
| R^2^ (within) | 0.47 | 0.46 | 0.52 |

*Note*: Robust standard errors are shown in brackets and are clustered at the district level.

Source: SUSENAS household surveys.

**^†^***p* < .10; **p* < .05; ***p* < .01. ^1)^ Age 15 – 49.

Table S11 Impact of electrification on fertility, and the role of media, 1993-1998, fixed effects regressions

|  |  |  | Contraception used | | Fertility ^1^ | | |
| --- | --- | --- | --- | --- | --- | --- | --- |
|  | TV | Newspaper | All | Traditional | (age 15 – 49) | | |
|  | (1) | (2) | (3) | (4) | (5) | (6) | (7) |
| Electricity coverage | 0.1848** | -0.0518* | 0.0161 | -0.0039 |  | -0.1030+ | -0.1227* |
|  | [0.0400] | [0.0222] | [0.0187] | [0.0030] |  | [0.0578] | [0.0597] |
| Watch TV |  |  | 0.1176** | 0.0033 | -0.0948* | -0.0739+ |  |
|  |  |  | [0.0150] | [0.0021] | [0.0455] | [0.0440] |  |
| Listen to radio |  |  | -0.0165 | -0.0013 | 0.0914 | 0.0878 |  |
|  |  |  | [0.0143] | [0.0027] | [0.0582] | [0.0571] |  |
| Read newspaper |  |  | -0.1496** | -0.0013 | -0.0868 | -0.0964 |  |
|  |  |  | [0.0273] | [0.0037] | [0.0833] | [0.0831] |  |
| Contraceptives used |  |  |  |  | -0.1031 | -0.1042 | -0.1183 |
|  |  |  |  |  | [0.0894] | [0.0888] | [0.0877] |
| Traditional contraceptives |  |  |  |  | 0.0126 | -0.0268 | -0.0710 |
| used |  |  |  |  | [0.5689] | [0.5702] | [0.5765] |
| Rural population share | 0.1564+ | 0.0872 | -0.0664+ | -0.0002 | 0.3282** | 0.3017** | 0.3048** |
|  | [0.0853] | [0.0780] | [0.0351] | [0.0039] | [0.0740] | [0.0734] | [0.0732] |
| Composition of female pop.  (ref: share aged 15-19) |  |  |  |  |  |  |  |
| Share aged 20-24 | -0.2494 | 0.0672 | 0.2268* | -0.0179 | 0.0979 | 0.0774 | 0.1000 |
|  | [0.1821] | [0.1211] | [0.0997] | [0.0132] | [0.2227] | [0.2234] | [0.2242] |
| Share aged 25-29 | -0.2207 | 0.0599 | 0.5087** | -0.0115 | 0.1773 | 0.1373 | 0.1484 |
|  | [0.1805] | [0.0990] | [0.0844] | [0.0105] | [0.2345] | [0.2326] | [0.2300] |
| Share aged 30-34 | -0.3105+ | -0.0115 | 0.5347** | -0.0029 | 0.1264 | 0.1339 | 0.1351 |
|  | [0.1860] | [0.1164] | [0.0937] | [0.0128] | [0.2228] | [0.2231] | [0.2217] |
| Share aged 35-39 | -0.5027* | 0.0325 | 0.4281** | -0.0002 | 0.1339 | 0.1043 | 0.1429 |
|  | [0.2378] | [0.1310] | [0.1051] | [0.0131] | [0.2730] | [0.2673] | [0.2720] |
| Share aged 40-44 | 0.0507 | 0.0876 | 0.4755** | -0.0159 | 0.2951 | 0.2656 | 0.2660 |
|  | [0.2247] | [0.1434] | [0.1152] | [0.0165] | [0.3112] | [0.3067] | [0.3094] |
| Share aged 45-49 | -0.6268* | -0.2466+ | 0.3792** | 0.0078 | -0.2358 | -0.2649 | -0.2153 |
|  | [0.2664] | [0.1335] | [0.1187] | [0.0145] | [0.3022] | [0.3011] | [0.3025] |
| Highest education completed by women aged 15-49 (ref: none) |  |  |  |  |  |  |  |
| Primary | 0.1091 | -0.1390** | 0.1186** | 0.0120 | -0.0624 | -0.0345 | -0.0285 |
|  | [0.0888] | [0.0510] | [0.0358] | [0.0073] | [0.1194] | [0.1207] | [0.1217] |
| Junior secondary | 0.5119** | 0.3693** | 0.1224+ | 0.0018 | -0.1454 | -0.0868 | -0.1589 |
|  | [0.1447] | [0.1144] | [0.0707] | [0.0106] | [0.1914] | [0.1938] | [0.2039] |
| Senior secondary | 0.3933* | 0.6495** | -0.0389 | 0.0008 | 0.1559 | 0.1723 | 0.1099 |
|  | [0.1543] | [0.1293] | [0.0691] | [0.0145] | [0.1719] | [0.1727] | [0.1662] |
| Higher | 0.9112* | 1.0205** | -0.2048 | 0.0248 | 0.7915+ | 0.6813 | 0.5960 |
|  | [0.3920] | [0.2740] | [0.1687] | [0.0224] | [0.4288] | [0.4327] | [0.4485] |
| Log per cap. exp. (t-1) | 0.0603* | 0.0187 | 0.0046 | 0.0018 | 0.0032 | 0.0161 | 0.0098 |
|  | [0.0287] | [0.0186] | [0.0132] | [0.0017] | [0.0359] | [0.0356] | [0.0351] |
| Constant | -0.0920 | -0.1175 | -0.0953 | -0.0128 | 1.8028** | 1.7350** | 1.8014** |
|  | [0.3669] | [0.2417] | [0.1714] | [0.0202] | [0.4313] | [0.4281] | [0.4209] |
| Year dummy variables | Yes | Yes | Yes | Yes | Yes | Yes | Yes |
| Number of observations | 1305 | 1305 | 1305 | 1305 | 1566 | 1566 | 1566 |
| Number of districts | 261 | 261 | 261 | 261 | 261 | 261 | 261 |
| R^2^ (within) | 0.91 | 0.64 | 0.78 | 0.09 | 0.41 | 0.41 | 0.41 |

*Note*: Robust standard errors are shown in brackets and are clustered at the district level.

Source: SUSENAS household surveys.

**^†^***p* < .10; **p* < .05; ***p* < .01. ^1)^ Age 15 – 49.

Table S12 Impact of electrification on fertility (average number of live births), women 15 to 49, DHS, 1991-2007, Poisson model

|  | Fertility | Fertility |
| --- | --- | --- |
|  | (1) | (2) |
| Electricity coverage | -0.092** | -0.025** |
|  | [0.006] | [0.005] |
| Composition of female population  (ref: share aged 15-19) |  |  |
| Share aged 20-24 |  | 0.247** |
|  |  | [0.012] |
| Share aged 25-29 |  | 0.626** |
|  |  | [0.012] |
| Share aged 30-34 |  | 0.950** |
|  |  | [0.012] |
| Share aged 35-39 |  | 1.176** |
|  |  | [0.012] |
| Share aged 40-44 |  | 1.327** |
|  |  | [0.013] |
| Share aged 45-49 |  | 1.431** |
|  |  | [0.013] |
| Highest education completed by women aged 15-49 (ref: none) |  |  |
| Primary education |  | 0.029** |
|  |  | [0.007] |
| Secondary education |  | -0.054** |
|  |  | [0.008] |
| Education higher than secondary |  | -0.224** |
|  |  | [0.011] |
| Contraception used |  | -0.069** |
|  |  | [0.004] |
| Traditional contraceptive used |  | 0.021** |
|  |  | [0.010] |
| Asset index |  | -0.067** |
|  |  | [0.002] |
| Rural area |  | -0.055** |
|  |  | [0.005] |
| Constant | 1.069** | 0.118** |
|  | [0.004] | [0.015] |
| DHS wave dummies | No | Yes |
| Number of observation | 131,409 | 131,409 |

*Note*: Robust standard errors, i.e. corrected for intra-cluster correlation, in brackets.

*Source*: DHS, various years.

**^†^***p* < .10; **p* < .05; ***p* < .01.

Table S13 Impact of electrification on fertility preferences and the role of media, 1991-2007, DHS, Poisson model

|  | Number of desired children | |
| --- | --- | --- |
|  | (1) | (2) |
| Electricity coverage | -0.047** | -0.053** |
|  | [0.005] | [0.0052] |
| Watch TV |  | -0.024** |
|  |  | [0.0047] |
| Composition of female population  (ref: share aged 15-19) |  |  |
| Share aged 20-24 | 0.039** | 0.038** |
|  | [0.0126] | [0.0128] |
| Share aged 25-29 | 0.110** | 0.109** |
|  | [0.0127] | [0.0126] |
| Share aged 30-34 | 0.176** | 0.175** |
|  | [0.0127] | [0.0127] |
| Share aged 35-39 | 0.229** | 0.228** |
|  | [0.0129] | [0.0129] |
| Share aged 40-44 | 0.276** | 0.274** |
|  | [0.0132] | [0.0132] |
| Share aged 45-49 | 0.321** | 0.319** |
|  | [0.0134] | [0.0134] |
| Hh living in rural areas | -0.0146^+^ | -0.014** |
|  | [0.0044] | [0.0044] |
| Highest education completed by women aged 15-49 (ref: none) |  |  |
| Primary education | -0.039** | -0.041** |
|  | [0.0070] | [0.0070] |
| Secondary education | -0.065** | -0.068** |
|  | [0.0077] | [0.0077] |
| Higher | -0.084** | -0.086** |
|  | [0.0110] | [0.0109] |
| Asset index | -0.065** | -0.069** |
|  | [0.0023] | [0.0025] |
| Constant | 1.053** | 1.050** |
|  | [0.0148] | [-0.0149] |
| DHS wave dummies | Yes | Yes |
| Number of observation | 107,137 | 107,137 |

*Note*: Robust standard errors, i.e. corrected for intra-cluster correlation, in brackets.

*Source*: DHS, various years.

**^†^***p* < .10; **p* < .05; ***p* < .01.

Table S14 Impact of electrification on fertility and fertility preferences, and the role of child mortality, 1991-2007, DHS, Poisson model

|  | Mortality | Fertility | Desired fertility |
| --- | --- | --- | --- |
|  | (1) | (2) | (3) |
| Electricity coverage | -0.089** | -0.008 | -0.031** |
|  | [0.009] | [0.006] | [0.004] |
| Average cluster mortality |  | 0.456** | 0.393** |
|  |  | [0.005] | [0.006] |
| Composition of female population  (ref: share aged 15-19) |  |  |  |
| Share aged 15-24 | 0.056** | 0.257** | 0.041** |
|  | [0.011] | [0.012] | [0.012] |
| Share aged 25-29 | -0.060 | 0.623** | 0.110** |
|  | [0.011] | [0.012] | [0.012] |
| Share aged 30-34 | -0.302** | 0.949** | 0.178** |
|  | [0.013] | [0.012] | [0.012] |
| Share aged 35-39 | -0.655** | 1.189** | 0.236** |
|  | [0.015] | [0.012] | [0.012] |
| Share aged 40-44 | -1.345** | 1.344** | 0.290** |
|  | [0.024] | [0.013] | [0.012] |
| Share aged 45-49 | -2.573** | 1.464** | 0.345** |
|  | [0.044] | [0.013] | [0.013] |
| Hh living in rural areas | -0.087** | -0.039** | 0.002 |
|  | [0.009] | [0.005] | [0.004] |
| Highest education completed by women aged 15-49 (ref: none) |  |  |  |
| Primary education | 0.031 | 0.026** | -0.040** |
|  | [0.0166] | [0.007] | [0.006] |
| Secondary education | 0.190** | -0.069** | -0.077** |
|  | [0.009] | [0.008] | [0.007] |
| Education higher than secondary | 0.190** | -0.252** | -0.109** |
|  | [0.017] | [0.011] | [0.010] |
| Contraception used |  | 0.095** |  |
|  |  | [0.004] |  |
| Traditional contraceptive used |  | -0.001 |  |
|  |  | [0.010] |  |
| Asset index | -0.137** | -0.042** | -0.041** |
|  | [0.004] | [0.002] | [0.002] |
| DHS wave dummies | Yes | Yes | Yes |
| Number of observation | 131,409 | 131,409 | 107,137 |
| Constant | 0.194** | -0.262** | 0.730** |
|  | [0.021] | [0.016] | [0.015] |

*Note*: Robust standard errors, i.e. corrected for intra-cluster correlation, in brackets.

*Source*: DHS, various years.

**^†^***p* < .10; **p* < .05; ***p* < .01.
